# Hyperphosphatemic Familial Tumoral Calcinosis in Two Siblings with a Novel Mutation in *GALNT3* Gene: Experience from Southern Turkey

**DOI:** 10.4274/jcrpe.galenos.2018.2018.0134

**Published:** 2019-02-20

**Authors:** Rabia Miray Kışla Ekinci, Fatih Gürbüz, Sibel Balcı, Atıl Bişgin, Mehmet Taştan, Bilgin Yüksel, Mustafa Yılmaz

**Affiliations:** 1 Çukurova University Faculty of Medicine, Department of Pediatric Rheumatology, Adana, Turkey; 2 Çukurova University Faculty of Medicine, Department of Pediatric Endocrinology, Adana, Turkey; 3 Çukurova University Faculty of Medicine, Department of Medical Genetics, Adana, Turkey

**Keywords:** GALNT3, hyperphosphatemia, tumoral calcinosis

## Abstract

Inactivating autosomal recessive mutations in fibroblast growth factor 23 *(FGF23), klotho (KL)* and *polypeptide N-acetylgalactosaminotransferase* 3 (*GALNT3*) genes lead to a rare disorder, hyperphosphatemic familial tumoral calcinosis (HFTC). Patients with HFTC present with hyperphosphatemia and tumor like soft tissue calcifications. Although 78% of patients develop their first symptoms between the ages of 2-13 years, diagnosis is usually delayed until adulthood. Some individuals with the same genetic defect develop a condition named hyperphosphatemic hyperostosis syndrome. Herein we report two siblings suffering from periarticular, warm, hard and tender subcutaneous masses. Subcutaneous calcifications were present on X-ray and biopsy results were consistent with calcinosis in both patients. Laboratory results showed marked hyperphosphatemia and elevated renal tubular phosphate reabsorption rates, normal renal function tests and normal serum 25-hydroxyvitamin D levels. Thus, we suspected HFTC and performed next generation sequencing for the *GALNT3* gene, reported as the most frequent cause. A novel homozygote P85Rfs*6 (c.254_255delCT) mutation in *GALNT3* was identified in both siblings. Our report adds two new patients to the literature about this rare genetic disease and suggests that small deletions in the *GALNT3* gene may be related with HFTC phenotype.

**What is already known on this topic?**

Mutations in the *FGF23, KL* and *GALNT3* genes cause hyperphosphatemic familial tumoral calcinosis (HFTC), which is a rare disorder. Patients with HFTC commonly present with hyperphosphatemia and tumor-like soft tissue calcifications. The main management strategy for HFTC is pain control and phosphate depletion.

**What this study adds?**

We describe two siblings with hyperphosphatemic familial tumoral calcinosis due to a novel homozygote *GALNT3* mutation and add to the scarce literature. We wish to emphasize that physicians should also consider this rare condition in the differential diagnosis of calcinosis.

## Introduction

Hyperphosphatemic familial tumoral calcinosis (HFTC) is a very rare disorder of phosphate homeostasis resulting from decreased fibroblast growth factor 23 (FGF23) synthesis or activity (1). *FGF23* gene encodes this protein which inhibits the sodium phosphate cotransporter in proximal renal tubules and 25-hydroxyvitamin D 1-α-hydroxylase expression, by its co-receptor *klotho (KL)*. The *polypeptide N-acetylgalactosaminotransferase 3 (GALNT3)* gene codes the enzyme known variously as UDP-N-acetyl-alpha-D galactosamine or polypeptide N-acetylgalactosaminyltransferase-3 (GalNAc-T3), which protects intact *FGF23* from catabolism and inactivation by posttranslational glycosylation (2). Inactivating autosomal recessive mutations in *FGF23*, *KL* or the *GALNT3* genes lead to increased renal tubular phosphate reabsorption and, usually, elevated 1,25-dihydroxyvitamin D_3_ (1,25-OH_2_D_3_), promoting gastrointestinal absorption of calcium and phosphorus (1,3,4). 

Patients with HFTC usually present with hyperphosphatemia and tumor-like soft tissue calcifications. Although 78% of patients develop their first symptoms between two and 13 years of age, diagnosis is usually delayed until adulthood. Some individuals with the same genetic defect develop hyperphosphatemic hyperostosis syndrome (HHS), a condition which was formerly described as a distinct entity (5). Here we report childhood onset HFTC in two siblings with a novel homozygote *GALNT3* mutation.

## Case Reports

### Case 1

A previously healthy 10 year-old female patient presented with complaints of pain and swelling in her left elbow. Due to the limitation of movement of the elbow, surgery was performed in another medical center at the age of eight years. Excisional biopsy revealed well-circumscribed subcutaneous tissue including widespread dystrophic calcification and multinuclear giant cells. She was referred to us upon recurrence of bilateral calcinosis in her elbows and in her right upper thigh. 

**Figure 1:**
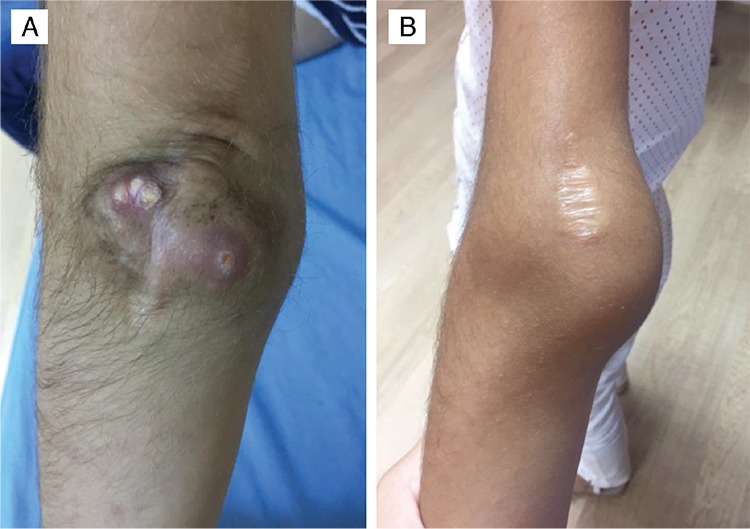
A) Calcinosis in the left elbow of Case 1. B) Subcutaneous mass around the left elbow of Case 2

**Figure 2:**
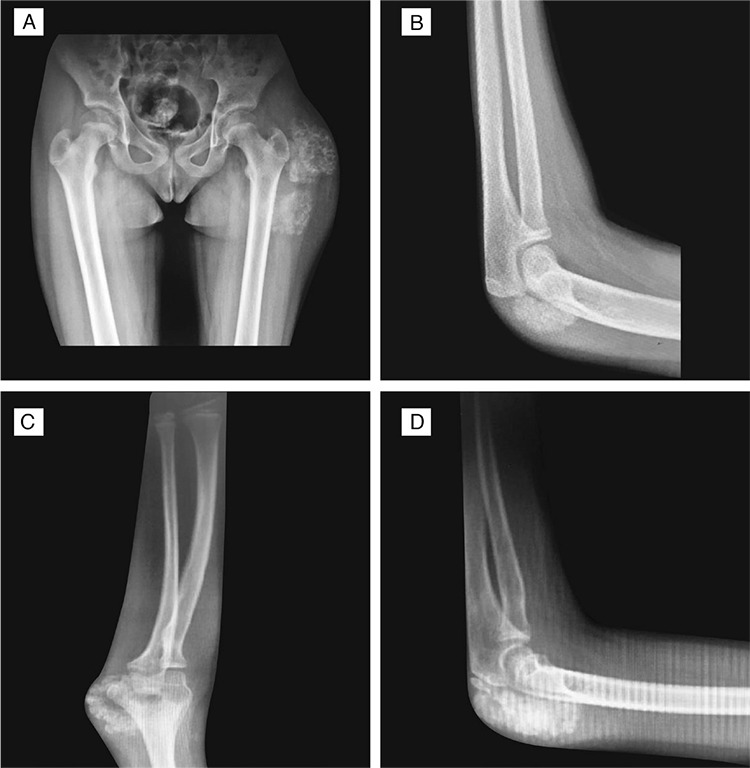
Radiographic findings in our two patients. Two giant radio-opaque soft tissue masses around the left femur neck of Case 1 in anteroposterior view (A). Lateral view of the left elbow of Case 1, revealing subcutanous calcifications (B). Anteroposterior view of upper extremities shows a radio-opaque mass around the elbow joint of Case 2 (C). A subcutaneous calcified mass behind the olecranon is identified in a lateral view of the left elbow (D)

The patient was the offspring of a first-degree cousin marriage. Her past medical history revealed no myositis, skin lesions or renal disease. Physical examination revealed calcinous masses of approximately 3 cm-6 cm diameters in the left elbow, the right elbow and in the right upper thigh (Figure 1). The masses were warm, hard and tender. Laboratory results showed marked hyperphosphatemia, normal serum creatinine, 25-hydroxyvitamin D and parathormone levels and an elevated ratio of tubular maximum reabsorption of phosphorus/glomerular filtration rate (TmP/GFR), consistent with HFTC (Table 1). Direct radiographs demonstrated radio-opaque soft tissue masses around the elbows bilaterally and right upper femur diaphysis (Figure 2). Bone mineral density Z-score was 0.7. Dental and ophthalmological examination showed no involvement. Milimetric calcified plaques were present inside the right lower eyelid. A novel homozygote P85Rfs*6 (c.254_255delCT) mutation in exon 1 of the *GALNT3* gene was detected by next generation sequencing (NGS). *In silico* analyses was performed with Mutation Taster, which confirmed that the mutation led to frameshift and a premature stop codon. Both parents were heterozygous carriers for the same mutation.

### Case 2

This nine year-old female patient was simultaneously referred to our department with her older sister, Case 1. She had developed similar but milder complaints over the preceding two years, including swelling of the left elbow which required surgery due to joint contracture and bilateral recurrence in her elbows thereafter. Direct radiographs demonstrated radio-opaque soft tissue masses around both elbows (Figure 2). Dental and ophthalmological examination showed no involvement. Hyperphosphatemia, elevated TmP/GFR ratio, family history, biopsy result and presence of the same homozygote P85Rfs*6 (c.254_255delCT) mutation in *GALNT3* gene confirmed the diagnosis of HFTC. 

**Figure 3:**
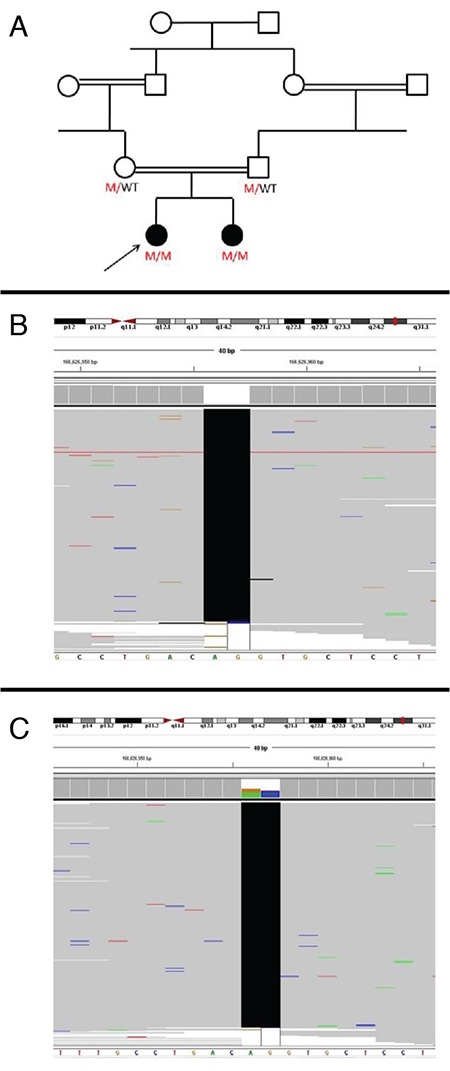
Genetic pedigree of the hyperphosphatemic familial tumoral calcinosis patients is presented in A. Next generation sequence view of the variant identified in the two cases are shown in B (Case 1) and C (Case 2)

Figure 3 shows the pedigree and NGS results of our patients. 

A written informed consent for this report was obtained from the parents of the patients.

## Discussion

Tumoral calcinosis (TC) is a condition in which calcium crystals accumulate in soft tissues, particularly in periarticular regions. HFTC is the autosomal recessive inherited form of TC with hyperphosphatemia and normal renal function. Differential diagnosis includes chronic renal failure, hypervitaminosis D, primary hyperparathyroidism or connective tissue diseases with particular emphasis on dermatomyositis and scleroderma. HFTC is very rare, approximately 75 cases have been genetically described worldwide to the best of our knowledge and almost all of the information is based on case reports (5,6,7,8,9,10). Homozygote mutations in the *GALNT3*, *FGF23* and *KL* genes were found in patients with the HHS phenotype. HHS is characterized by painful diaphyseal hyperostosis and may overlap with the TC phenotype in some cases. One study speculated that nonsense and missense *GALNT3* mutations are associated with TC and HHS phenotypes, respectively (11). Indeed, the majority of reported *GALNT3* mutations are missense or nonsense, and only five distinct small deletions were identified in HFTC patients, according to The Human Gene Mutation Database. Small deletions were reported to cause only the TC phenotype, as was also true for our patients (8,12,13).

Besides subcutaneous calcifications, patients often present with dental abnormalities and occasionally anemia, low-grade fever, regional lymphadenopathy, splenomegaly, amyloidosis, chronic recurrent osteomyelitis and eyelid calcifications (14,15,16). Vascular calcifications may rarely occur and can cause significant morbidity (17). Some HFTC patients develop hyperphosphatemia several years after the onset of dental abnormalities and calcinosis (11). Eyelid calcification was present in one of our patients. However, other clinical traits had not yet developed at presentation.

The management of HFTC mainly targets pain control and phosphate depletion. Surgery is not recommended, due to recurrences, until the calcinosis causes restricted joint movement. A phosphate restricted diet and phosphate binders are the mainstays of the medical treatment (18). A calcium-free phosphate binder, Sevelamer alters the intestinal absorption of phosphorus. Although Sevelamer and dietary phosphate restriction is reported to lead to complete or partial recovery of the calcinosis, recurrences have been reported due possibly to self-discontinuation or ineffectiveness of the drug in a significant proportion of patients. Other agents, including acetazolamide, probenocid and topical sodium thiosulfate, have been reported to be beneficial with variable outcomes (8,19,20,21).

The limitation of our study was the unavailability of serum 1,25-OH_2_D_3_ level determinations in both our patients. However, the patients had other clinical and laboratory results consistent with HFTC diagnosis. We believe that elevated serum 1,25-OH_2_D_3_ levels are only supportive in the diagnosis of HFTC. 

In conclusion, we report two siblings with a novel homozygote *GALNT3* mutation representing an HFTC phenotype. HFTC is a rare cause of tenderness and pain around the joints in children and should be kept in mind in the differential diagnosis of arthritis. Our report adds two new patients to the information on a rare genetic disease and we wish to highlight the need for attention to this rare disorder. We speculate that small deletions in *GALNT3* gene may be related with HFTC phenotype. However, this speculation can be confirmed only with genotype-phenotype correlation studies including long-term outcomes of more patients in the future.

## Figures and Tables

**Table 1:**
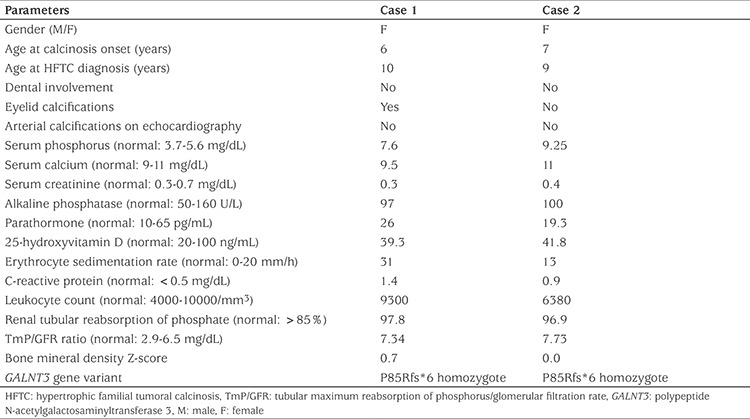
Clinical characteristics, laboratory and genetic results of the two patients with hypertrophic familial tumoral calcinosis
